# Red Blood Cell Transfusion and Postoperative Delirium in Hip Fracture Surgery Patients: A Retrospective Observational Cohort Study

**DOI:** 10.1155/2021/8593257

**Published:** 2021-11-22

**Authors:** Jacob Raphael, Nadia B. Hensley, Jonathan Chow, K. Gage Parr, John S. McNeil, Steven B. Porter, Monica Taneja, Kenichi Tanaka, Michael Mazzeffi

**Affiliations:** ^1^University of Virginia School of Medicine, Charlottesville, VA, USA; ^2^Johns Hopkins University School of Medicine, Baltimore, MD, USA; ^3^George Washington University School of Medicine and Health Sciences, Washington, DC, USA; ^4^Mayo Clinic Alix School of Medicine, Jacksonville, FLA, USA; ^5^University of Maryland School of Medicine, Baltimore, MD, USA; ^6^Oklahoma University School of Medicine, Oklahoma, OK, USA

## Abstract

**Background:**

Patients having hip fracture surgery are at high risk for postoperative delirium. Red blood cell (RBC) transfusion may increase postoperative delirium risk by causing neuroinflammation. We hypothesized that RBC transfusion would be associated with postoperative delirium in patients having hip fracture surgery.

**Methods:**

An observational cohort study was performed using the United States National Surgical Quality Improvement Program (NSQIP) participant use files for hip fracture from 2016 to 2018. Propensity score analysis and inverse probability of treatment weighting (IPTW) were used to reduce bias from confounding. An IPTW adjusted odds ratio for developing postoperative delirium was calculated for patients who received RBC transfusion during surgery or in the 72 hours after.

**Results:**

There were 20,838 patients who had eligible current procedural terminology (CPT) codes for primary hip fracture surgery and complete study data. After employing strict exclusions to balance covariates and reduce bias, 3,715 patients remained in the IPTW cohort. Of these, 626 patients (16.9%) received RBC transfusion and 665 patients (17.9%) developed postoperative delirium. IPTW adjustment led to good covariate balance between patients who received RBC transfusion and those who did not. Patients who received RBC transfusion had significantly higher odds of postoperative delirium, IPTW adjusted odds ratio = 1.21, 95% CI = 1.03 to 1.43, and *P* = 0.02. Discharge location also differed significantly between patients who received RBC transfusion and those who did not (*P* < 0.001) with in-hospital mortality or referral to hospice occurring in 1.6% of patients who received RBC transfusion and 1.3% of patients who were not transfused.

**Conclusion:**

RBC transfusion is associated with increased odds of postoperative delirium after hip fracture surgery and may be associated with worse clinical outcome.

## 1. Introduction

Hip fractures are an important cause of morbidity and mortality in geriatric patients. Over the next decade, the number of hip fracture surgeries per year in the United States is expected to increase by 12%, up to 300,000 surgeries per year [1]. Patients having hip fracture surgery commonly have comorbidities that increase their risk for cardiopulmonary, thromboembolic, and neurocognitive complications after surgery. In-hospital and 30-day mortality are high and may exceed 10% in this high-risk patient population [2].

Patients undergoing hip fracture surgery have a high incidence of perioperative anemia and frequently require red blood cell (RBC) transfusion [3]. In a retrospective cohort study that included 835 patients undergoing hip fracture surgery, Desai et al. reported that 40% of patients received RBC transfusion [4]. RBCs are transfused to increase oxygen delivery, particularly in patients with poor cardiopulmonary reserve and/or preexisting anemia. However, RBC transfusion also increases the risk for complications after hip fracture surgery, and the optimal threshold for RBC transfusion in elderly high-risk patients is not known [5–7].

Delirium is a common and serious postoperative complication in patients undergoing hip fracture surgery with an incidence of between 13% and 70% in various studies [8, 9]. Postoperative delirium after hip fracture surgery is associated with increased morbidity, prolonged hospitalization, more frequent discharge to a long-term care facility, and increased mortality [10, 11].

It has been suggested that RBC transfusion increases postoperative delirium risk in cardiac surgical patients [12] and other critically ill patients [13, 14]. The purpose of our study was to investigate whether RBC transfusion is associated with an increased postoperative delirium risk in patients undergoing hip fracture surgery. We hypothesized that RBC transfusion (during surgery or in the first 72 hours after) would be associated with increased postoperative delirium risk.

## 2. Methods

### 2.1. Patients

The Institutional Review Board at the University of Maryland, Baltimore, approved the study and granted a waiver of written informed consent (HP-00084608). Adults who had hip fracture surgery were identified using the National Surgical Quality Improvement Program (NSQIP) participant use files from 2016 to 2018. NSQIP PUFs were used for the study because they contain granular data on demographics, comorbidities, and surgical details allowing for robust risk adjustment. Patients with the following current procedural terminology (CPT) codes were included: 27236 (open treatment of femoral fracture, proximal end, neck, internal fixation, or prosthetic replacement), 27244 (treatment of intertrochanteric, peritrochanteric, or subtrochanteric femoral fracture with plate/screw-type implant), and 27245 (treatment of intertrochanteric, peritrochanteric, or subtrochanteric femoral fracture with intramedullary implant). Study exclusions were as follows: preoperative delirium, age less than 65 or greater than 90, platelet count less than 50,000 × 10^9^ per uL, hematocrit less than 33% or greater than 45%, international normalized ratio (INR) greater than 1.5, body weight less than 40 kg or greater than 100 kg, and operative time less than one hour or greater than four hours. Strict exclusions were employed to eliminate the impact of severe outliers and increase the likelihood of good covariate balance between patients who did and did not receive RBC transfusion. Patients were excluded from the cohort if they were missing data for any variable included in the propensity score model.

### 2.2. Study Data

Demographics, medical comorbidities, and operative details were collected for patients in the cohort. Definitions for study variables were based on NSQIP definitions (https://www.facs.org). The following variables were collected: age, sex, height, weight, preoperative hematocrit, preoperative platelet count, preoperative creatinine, preoperative international normalized ratio, baseline functional status, American Society of Anesthesiologists (ASA) physical status classification, diabetes mellitus, hypertension requiring medication, congestive heart failure, tobacco use, chronic obstructive pulmonary disease, dialysis dependent, disseminated cancer, wound infection prior to surgery, steroid use, bleeding disorder history, RBC transfusion in the 72 hours before surgery, anesthesia type, CPT code, NSQIP predicted probability of morbidity, NSQIP predicted probability of mortality, emergency status, and total operative minutes.

### 2.3. Exposure

The study exposure was RBC transfusion during surgery or in the 72 hours after surgery, as defined by NSQIP. NSQIP does not record the exact timing of RBC transfusion, only the day of transfusion, and there are no data on non-RBC transfusion (such as platelets, fresh frozen plasma, or cryoprecipitate) or the total number of RBC units transfused.

### 2.4. Outcomes

The study's primary outcome was postoperative delirium, which NSQIP defines as an acute confusion state occurring in the 30 days after surgery. The details of how delirium is identified by NSQIP data collectors have been previously described in the published literature [11]. Secondarily, we collected data on hospital length of stay and discharge location.

### 2.5. Statistical Analysis

Statistical analysis was performed using SAS 9.3 (SAS Corporation, Cary, NC, USA). The analysis plan and methodology were checked against the strengthening and the reporting of observational studies in epidemiology (STROBE) checklist for cohort studies. The full cohort, which included all patients with eligible CPTs who had complete study data, was analyzed. After stratification by RBC transfusion, continuous variables were summarized as mean ± standard deviation, and categorical variables were summarized as the number and percentage of patients. Covariate balance was assessed between patients who received RBCs and those who did not by calculating standardized mean differences (SMDs). SMDs greater than 0.2 were considered to represent significant covariate imbalances. The unadjusted rate of postoperative delirium was compared between patients who received RBCs and those who did not, and an odds ratio (OR) was calculated with a 95% confidence interval.

After employing study exclusions to create a more homogeneous cohort, we fitted a nonparsimonious propensity score model with RBC transfusion modeled as the dependent variable. Independent variables that were included in the model included demographics, comorbidities, and operative details that might impact the risk of RBC transfusion or postoperative delirium. These variables included age, sex, weight, height, preoperative hematocrit, preoperative platelet count, preoperative creatinine, diabetes mellitus, hypertension requiring medication, congestive heart failure, tobacco use, chronic obstructive pulmonary disease, dialysis dependent, disseminated cancer, wound infection prior to surgery, steroid use, bleeding disorder history, emergency status, anesthesia type, CPT code, and RBC transfusion in the 72 hours before surgery.

Using calculated propensity scores, inverse probability of treatment weights (IPTWs) were calculated for each patient, and an IPTW adjustment was used to balance covariates between patients who received RBC transfusion and those who did not. IPTW allows for estimation of the causal effect when measured covariates are balanced between groups [15]. It also allows for effect estimation in the entire cohort rather than only in the exposed. For the IPTW cohort, continuous variables were summarized as mean ± standard deviation and categorical variables were summarized as the number and percentage of patients. Covariate balance was assessed between patients who received RBCs and those who did not by calculating SMDs. SMDs greater than 0.2 were considered to represent significant covariate imbalances. To calculate the average treatment effect of RBC transfusion on postoperative delirium, IPTW adjusted logistic regression was performed. An OR for postoperative delirium with a 95% confidence interval was calculated.

Several sensitivity analyses were also performed. First, we fit a multivariable logistic regression model using the full cohort with postoperative delirium modeled as the dependent variable. For this model, we performed stepwise logistic regression. A *P* value < 0.1 was required for model entry, and a *P* value < 0.05 was required for retainment in the final model. Independent variables that were considered for model entry included demographics, comorbidities, operative details, and RBC transfusion. The *c* statistic was reported for the final model, as a measure of discrimination, and appropriate model diagnostics including goodness-of-fit testing and multicollinearity analysis were performed. Odds ratios with 95% confidence intervals were reported for all independent variables in the model. Finally, we tested for effect modification from preoperative anemia. Specifically, we entered interaction terms for RBC transfusion and preoperative hematocrit <30% and RBC transfusion and preoperative hematocrit <24% into the model and evaluated their significance. A priori sample size calculation was not performed.

## 3. Results

**Figure 1:**
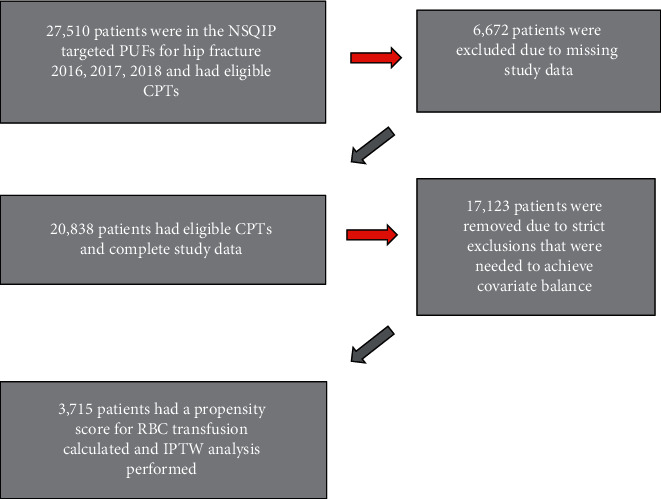
Flow diagram for the study. CPT = current procedural terminology, IPTW = inverse probability of treatment weighting, NSQIP = National Surgical Quality Improvement Program, PUF = participant use file, and RBC = red blood cell.

Figure 1 shows the flow diagram for the study. The NSQIP hip fracture PUFs from 2016 to 2018 contained a total of 31,751 patients. Of these, 27,510 had eligible CPTs and 20,838 had eligible CPTs and complete study data.  Table 1 lists characteristics of patients in the full cohort (*N* = 20,838). As indicated by high SMDs (>0.2), there was a significant imbalance in multiple variables including age, weight, preoperative hematocrit, preoperative creatinine, preoperative RBC transfusion, predicted morbidity, predicted mortality, ASA class, CPT code, and surgery duration.  Table 2 shows the crude rate of delirium and discharge location for patients in the full cohort. Postoperative delirium was significantly more common in patients who received RBC transfusion (22.2% vs. 17.9%, *P* < 0.001; OR = 1.31 (95% CI = 1.21 to 1.41)).

After employing strict exclusions to reduce bias, 3,715 patients were included in the propensity score calculation and IPTW analysis with 626 patients (16.9%) receiving RBC transfusion. Eighty-five percent of patients who received RBC transfusion received it during surgery or in the first 48 hours after.  Table 3 lists demographics, comorbidities, and operative details after IPTW adjustment. Study variables were well balanced between patients who did and did not receive RBC transfusion after IPTW (all SMDs <0.2). In the final IPTW cohort, the majority of patients were women, the mean age was 79 years, and the mean preoperative hematocrit was 37%. There was no difference in preoperative hematocrit between patients who received RBC transfusion and those who did not (37% ± 4 vs. 37% ± 2, respectively).

Table 4 shows outcomes in the IPTW cohort. The overall rate of postoperative delirium was 17.9%. Postoperative delirium was significantly more common in patients who received RBC transfusion (20.3% vs. 17.4%, *P* = 0.02). The IPTW adjusted OR for delirium in patients who received RBC transfusion was 1.21 (95% CI = 1.03 to 1.43,*P* = 0.02). The length of stay after surgery and discharge location differed significantly between patients who received RBC transfusion and those who did not (*P* < 0.001 for both comparisons). In-hospital mortality or referral to hospice occurred in 1.6% of patients who received RBC transfusion and in 1.3% of those who did not.

Supplementary Table 1 shows the results of the sensitivity analyses. After adjusting for multiple confounding variables in the full cohort, RBC transfusion remained significantly associated with postoperative delirium (OR = 1.21 (1.11 to 1.32, *P* < 0.001)). When the interaction between preoperative anemia (hematocrit <24% or hematocrit <30%) and RBC transfusion was tested in the model, there was no significant effect modification found (*P* = 0.46 and 0.48, respectively).

## 4. Discussion

In a retrospective observational cohort study of patients undergoing hip fracture surgery in the United States, we found that RBC transfusion was associated with increased odds of postoperative delirium even after IPTW. Patients who received RBC transfusion also had longer hospital stays and more frequently died or were discharged to hospice when compared to patients who did not receive RBC transfusion.

Preoperative anemia is common in elderly patients having hip fracture surgery. In a cohort study of 971 patients, the incidence of preoperative anemia was greater than 60%, with 40% of patients having hemoglobin less than 10.0 g/dL [16]. Anemia is a risk factor for postoperative morbidity, mortality, and unplanned hospital readmission [17]. RBCs are transfused to improve oxygen delivery and mitigate anemia's negative effects, but RBC transfusion is also associated with an increased risk of perioperative complications [5–7]. In a recent cohort study of 8,416 patients having hip fracture surgery, RBC transfusion was independently associated with 30-day mortality, hospital readmission, and increased length of hospital stay [18].

Several investigators have explored the association between RBC transfusion and postoperative delirium after hip fracture surgery, but results are inconsistent. Some studies have reported a positive association between RBC transfusion and delirium [19–21], while others found no association [11, 22, 23]. A recent meta-analysis that included 23 studies with 29,471 patients who were aged 55 or older did not find evidence of an association between RBC transfusion and delirium [24]. Many prior studies did not adequately control for confounding.

The pathophysiology of postoperative delirium is complex and multifactorial and includes factors such as older age, dementia, surgical trauma, and the use of anesthetic medications such as benzodiazepines and opioids. Uncontrolled postoperative pain also contributes to delirium. Several reports suggest that increased inflammation and oxidative stress play a role in the development of postoperative delirium [25, 26]. Tan et al. found that transfusion of older RBCs in an animal model was associated with cognitive impairment and increased neuroinflammation [27]. In human studies, there have been findings that suggest transfusion of older RBCs is associated with increased duration of delirium, increased levels of inflammatory markers, and increased lipid peroxidation byproducts [28]. Each unit of allogeneic RBCs contains a million donor leukocytes, even with leukoreduction, and the RBC supernatant contains over 400 proteins; some of which enhance inflammation, activate neutrophils, and impair innate immunity [29]. Interleukins (IL) 1 and 6 increase in the supernatant of stored RBCs and have been shown to cause neuroinflammation [30–32].

The optimal transfusion trigger in patients undergoing hip fracture surgery remains unclear. A large, prospective, randomized controlled trial that compared liberal transfusion (RBC transfusion to maintain a hemoglobin level of >10.0 g/dL) with restrictive transfusion (RBC transfusion to maintain a hemoglobin level of >8.0 g/dL) demonstrated no difference in mortality or the ability to ambulate without assistance 60 days after surgery. In addition, the incidence of other outcomes, such as 30- and 60-day mortality, acute coronary syndrome, and venous thromboembolism, was similar between groups [3]. Amin et al. [33] reported that a hemoglobin transfusion trigger as low as 7 g/dL, compared with a hemoglobin transfusion trigger of 8 g/dL, resulted in a significant decrease in cardiac morbidity and 30-day readmission in patients having hip fracture surgery. There was no difference in in-hospital morbidity or 90-day survival when using 7 g/dL as a hemoglobin transfusion trigger. Based on these data, as well as additional studies that investigated transfusion triggers in high-risk, elderly patients, recent guidelines recommend a hemoglobin transfusion trigger of 8.0 g/dL in orthopedic surgical patients [34–36]. The guidelines recognize, however, that a hemoglobin transfusion trigger of 7.0 g/dL is probably safe and comparable.

Beyond restrictive RBC transfusion, there are other patient blood management strategies that can help to reduce RBC transfusion in hip fracture surgery patients. Antifibrinolytic drugs, particularly tranexamic acid, have demonstrated efficacy in reducing bleeding and RBC transfusion in patients having hip fracture surgery and are not associated with an increased thrombotic risk [37]. Minimization of hemodilution and preoperative iron and erythropoietin treatment may further reduce RBC transfusion [38].

Our study has limitations. First, it is an observational cohort study that can only establish association, not causality. We were only able to control for observed confounders, and unobserved confounders may have created residual bias in our results. Second, since NSQIP does not record the exact timing of RBC transfusion, we could not confirm whether RBC transfusion preceded postoperative delirium in each individual case. Third, it is unclear whether RBC transfusion is a surrogate of surgical complexity, despite similar operative times between groups. Along these lines, there are no data on intraoperative blood loss in NSQIP. Fourth, our IPTW cohort had multiple exclusion criteria, and ultimately, less than 15% of patients in the NSQIP hip fracture PUFs were included in our analysis. This may limit the generalizability of our study's results, but it was necessary to achieve good covariate balance and reduce bias maximally. Fifth, the NSQIP definition for postoperative delirium is somewhat vague and is not based on a validated delirium assessment such as the Confusion Method Assessment (CAM) score. Sixth, NSQIP does not record information on prior cerebrovascular accidents, mini-mental status exam results, or information on hospitals, which would allow adjustment for hospital trends/patterns. Finally, data about the total number of RBC units and other blood products that were transfused were not available. This is important, as a prior study suggested that transfusion of more than 2 RBC units was associated with an increased delirium risk, but transfusion of 1 or 2 units was not [39].

## 5. Conclusion

In conclusion, in a retrospective observational cohort study of patients undergoing surgery for hip fracture, we found a significant independent association between RBC transfusion and postoperative delirium. Future multi-institutional studies are needed to further test this hypothesis. These studies should explore the pathophysiologic mechanisms that trigger neurological injury and delirium in patients who receive RBC transfusion and investigate the role of patient blood management, restrictive transfusion, and preoperative anemia treatment in decreasing delirium after hip fracture surgery.

## Figures and Tables

**Table 1:** Characteristics of all patients with eligible CPTs and complete study data.

Variable	No RBC transfusion	RBC transfusion^*∗*^	Standardized mean difference
	*N* = 15,311	*N* = 5,527	
Age (years)	78 ± 11	81 ± 10	0.29
Sex (% male)	5,078 (33.2)	1,437 (26.0)	0.16
Weight (kg)	69 ± 18	65 ± 17	0.23
Height (cm)	164 ± 11	162 ± 10	0.19
Hematocrit (%)	36 ± 5	31 ± 5	>0.99
Platelet count × 10^9^/L	210 ± 79	198 ± 81	0.15
Creatinine (mg/dL)	1.0 ± 0.8	1.2 ± 0.9	0.23
INR	1.1 ± 0.2	1.1 ± 0.3	<0.01
Independent functional status at baseline	12,637 (82.5)	4,353 (78.8)	0.09
Diabetes mellitus	2,880 (18.8)	1,062 (19.2)	0.01
Hypertension requiring medication	9,933 (64.9)	3,898 (70.5)	0.12
Congestive heart failure	471 (3.1)	229 (4.1)	0.05
Tobacco use	2,071 (13.5)	503 (9.1)	0.14
COPD	1,668 (10.9)	644 (11.7)	0.03
Dialysis	291 (1.9)	149 (2.7)	0.05
Dementia	3,161 (20.7)	1,269 (23.0)	0.06
Disseminated cancer	514 (3.4)	219 (4.0)	0.03
Wound infection	513 (3.4)	275 (5.0)	0.08
Steroid use	882 (5.8)	408 (7.4)	0.06
Bleeding disorder	2,398 (15.7)	1,221 (22.1)	0.16
Transfused in 72 hours before surgery	392 (2.6)	510 (9.2)	0.28
Emergency surgery	4,475 (29.2)	1,648 (29.8)	0.31
Mortality probability (%)	4.6 ± 5.7	6.6 ± 7.1	
Morbidity probability (%)	9.4 ± 4.5	10.8 ± 4.9	

ASA class			
I or II	3,014 (19.7)	681 (12.3)	0.30
III	9,496 (62.0)	3,536 (64.0)	0.04
IV or V	2,801 (18.3)	1,310 (23.7)	0.13

Anesthesia type			
General	10,828 (70.7)	4,217 (76.3)	0.13
Spinal	3,300 (21.6)	1,004 (18.2)	0.09
MAC and local	1,072 (7.0)	271 (4.9)	0.09
Regional	111 (0.7)	35 (0.6)	0.01

CPT code			
27236	6,243 (40.8)	1,191 (21.6)	0.42
27244	1,784 (11.7)	645 (11.7)	<0.01
27245	7,284 (47.5)	3,691 (66.8)	0.40
Duration of surgery (minutes)	64 ± 38	74 ± 48	0.23

ASA = American Society of Anesthesiologists, COPD = chronic obstructive pulmonary disease, CPT = current procedural terminology, INR = international normalized ratio, MAC = monitored anesthesia care, and RBC = red blood cell. CPT codes: 27236 (open treatment of femoral fracture, proximal end, neck, internal fixation, or prosthetic replacement), 27244 (treatment of intertrochanteric, peritrochanteric, or subtrochanteric femoral fracture with plate/screw-type implant), and 27245 (treatment of intertrochanteric, peritrochanteric, or subtrochanteric femoral fracture with intramedullary implant). Values are mean +sd or *n* (%). ^*∗*^Timing of RBC transfusion: postoperative day (POD), POD0 = 1,356, POD1 = 1,977, POD2 = 1,683, POD3 = 493, and others = 18.

**Table 2:** Outcomes for all patients with eligible CPTs and complete study data.

Variable	No RBC transfusion	RBC transfusion	*P* value
	*N* = 15,311	*N* = 5,527	
Delirium	2,746 (17.9)	1,228 (22.2)	<0.001
Length of stay after surgery (days)	6 ± 8	7 ± 9	<0.001

Discharge location			<0.001
Died or hospice	361 (2.3)	184 (3.3)	
Home	2,736 (17.9)	496 (9.0)	
Rehabilitation	4,237 (27.7)	1,797 (32.5)	
Other hospitals	473 (3.1)	167 (3.0)	
Nursing home	7,479 (48.8)	2,870 (52.0)	
No data	25 (0.2)	13 (0.2)	

Values are mean +sd or *n* (%). CPT = current procedural terminology; RBC = red blood cell.

**Table 3:** Characteristics of patients in IPTW cohort.

Variable	No RBC transfusion	RBC transfusion^*∗*^	Standardized mean difference
	*N* = 3,089	*N* = 626	
Age (years)^*∗∗*^	79 ± 5	79 ± 11	<0.01
Sex (% male)^*∗∗*^	856 (27.7)	170 (27.1)	0.01
Weight (kg)^*∗∗*^	68 ± 11	68 ± 24	<0.01
Height (cm)^*∗∗*^	164 ± 8	163 ± 16	0.08
Hematocrit (%)^*∗∗*^	37 ± 2	37 ± 4	<0.01
Platelet count *×* 10^9^/L^*∗∗*^	213 ± 54	211 ± 120	0.02
Creatinine (mg/dL)^*∗∗*^	1.0 ± 0.5	1.0 ± 1.1	<0.01
INR	0.8 ± 0.30	0.8 ± 0.6	<0.01
Independent functional status at baseline	2,619 (84.8)	531 (84.8)	<0.01
Diabetes mellitus^*∗∗*^	568 (18.4)	128 (20.5)	0.05
Hypertension requiring medication^*∗∗*^	2,036 (65.9)	416 (66.4)	0.01
Congestive heart failure^*∗∗*^	62 (2.0)	18 (2.8)	0.05
Tobacco use^*∗∗*^	327 (10.6)	62 (9.9)	0.02
COPD^*∗∗*^	327 (10.6)	71 (11.4)	0.03
Dialysis^*∗∗*^	40 (1.3)	9 (1.4)	<0.01
Dementia	602 (19.5)	128 (20.5)	0.03
Disseminated cancer^*∗∗*^	111 (3.6)	19 (3.1)	0.03
Wound infection^*∗∗*^	80 (2.6)	15 (2.4)	0.01
Steroid use^*∗∗*^	185 (6.0)	41 (6.6)	0.02
Bleeding disorder^*∗∗*^	374 (12.1)	83 (13.2)	0.03
Transfused in 72 hours before surgery ^*∗∗*^	22 (0.7)	5 (0.8)	0.01
Emergency surgery^*∗∗*^	775 (25.1)	197 (31.4)	0.14
Mortality probability (%)	3.4 ± 3.4	3.7 ± 6.7	0.06
Morbidity probability (%)	8.4 ± 3.0	9.0 ± 6.9	0.11

ASA class			
I or II	720 (23.3)	128 (20.5)	0.07
III	1,942 (62.9)	392 (62.6)	<0.01
IV or V	427 (13.8)	106 (16.9)	0.09

Anesthesia type^*∗∗*^			
General	2,215 (71.7)	457 (73.0)	0.03
Spinal	627 (20.3)	121 (19.3)	0.03
MAC and local	225 (7.3)	43 (6.9)	0.02
Regional	22 (0.7)	5 (0.8)	0.01

CPT code^*∗∗*^			
27236	1,751 (56.7)	348 (55.6)	0.02
27244	229 (7.4)	41 (6.5)	0.04
27245	1,109 (35.9)	237 (37.8)	0.04
Duration of surgery (minutes)	89 ± 20	91 ± 45	0.06

ASA = American Society of Anesthesiologists, COPD = chronic obstructive pulmonary disease, CPT = current procedural terminology, INR = international normalized ratio, IPTW = inverse probability of treatment weighting, MAC = monitored anesthesia care, and RBC = red blood cell. CPT codes: 27236 (open treatment of femoral fracture, proximal end, neck, internal fixation, or prosthetic replacement), 27244 (treatment of intertrochanteric, peritrochanteric, or subtrochanteric femoral fracture with plate/screw-type implant), and 27245 (treatment of intertrochanteric, peritrochanteric, or subtrochanteric femoral fracture with intramedullary implant). Values are mean +sd or *n* (%) ^*∗*^Timing of RBC transfusion: postoperative day (POD), POD0 = 121, POD1 = 178, POD2 = 234, POD3 = 90, and others = 3 ^*∗∗*^Variable was included in the propensity score model. Other variables were not included in the propensity score model, but their balance was assessed.

**Table 4:** Outcomes for patients in the IPTW cohort.

Variable	No RBC transfusion	RBC transfusion	*P* value^*∗*^
	*N* = 3,089	*N* = 626	
Delirium	537 (17.4)	127 (20.3)	0.02
Length of stay after surgery (days)	6 ± 5	7 ± 10	<0.001

Discharge location			<0.001
Died or hospice	40 (1.3)	10 (1.6)	
Home	581 (18.8)	96 (15.3)	
Rehabilitation	914 (29.6)	225 (36.0)	
Other hospitals	65 (2.1)	24 (3.9)	
Nursing home	1,486 (48.1)	271 (43.2)	
No data	3 (0.1)	0 (0)	

Values are mean +sd or *n* (%). IPTW = inverse probability of treatment weighting; RBC = red blood cell. ^*∗*^*P* values are weighted with IPTW.

## Data Availability

The data used for this study can be obtained from the United States National Surgical Quality Improvement Program Participant Use Files.

## Supplementary Materials

Supplementary Materials Supplementary table shows the results of the multivariable logistic regression model sensitivity analysis. .
