# Supplementary material for: Red Blood Cell Transfusion and Postoperative Delirium in Hip Fracture Surgery Patients: A Retrospective Observational Cohort Study
Source: Anesthesiol Res Pract. 2021 Nov 22;2021:8593257. doi: 10.1155/2021/8593257 (PMC8629661; doi:10.1155/2021/8593257)
Supplement: Supplementary Materials — Supplementary table shows the results of the multivariable logistic regression model sensitivity analysis. . [file 8593257.f1.docx]

Supplemental Table 1. Multivariable logistic regression model for postoperative delirium

| Variable | Odds ratio with 95% confidence interval | P value |
| --- | --- | --- |
| Age | 1.04 (1.04 to 1.05) | <0.001 |
| Female sex | 0.83 (0.77 to 0.90) | <0.001 |
| ASA 3 vs. ASA 1 or 2 | 1.71 (1.52 to 1.93) | <0.001 |
| ASA 4 or 5 vs. ASA 1 or 2 | 1.83 (1.57 to 2.13) | <0.001 |
| Smoker | 1.18 (1.05 to 1.34) | 0.007 |
| Disseminated cancer | 0.75 (0.60 to 0.94) | 0.01 |
| Infected wound | 1.41 (1.19 to 1.67) | <0.001 |
| Preoperative hematocrit (per 1% increase) | 1.02 (1.01 to 1.03) | <0.001 |
| Non-emergency surgery | 1.18 (1.09 to 1.28) | <0.001 |
| Predicted mortality (per 10% increase) | 1.29 (1.20 to 1.39) | <0.001 |
| RBC transfusion during surgery or first 72 hours after | 1.21 (1.11 to 1.32) | <0.001 |

Model was fit using stepwise logistic regression with a P value <0.1 used for model entry and <0.05 for retainment in the final model. Independent variables that were considered for the model inclusion were all demographics, comorbidities, operative details, and RBC transfusion.

The C statistic for the model was 0.65. The variance inflation factor and a correlation matrix were used to assess for multicollinearity. No significant multicollinearity was detected.

Interaction terms were tested for RBC transfusion and preoperative hematocrit <30% and RBC transfusion and preoperative hematocrit <24%. P values for the interaction terms were 0.46 and 0.48 respectively, indicating no effect modification.

ASA=American Society of Anesthesiologists physical status, RBC=red blood cell
